# The complete chloroplast genome sequences of *Barnardia japonica* (Thunb.) Schult. and Schult.f

**DOI:** 10.1080/23802359.2018.1481790

**Published:** 2018-06-22

**Authors:** Rui-Hong Wang, Jing Gao, Mengdi Li, Xue Wu, Chao Shen, Junjie Wu, Zhe-Chen Qi, Pan Li

**Affiliations:** a College of Life Sciences, Zhejiang Sci-Tech University, Hangzhou, China;; b Zhejiang Province Key Laboratory of Plant Secondary Metabolism and Regulation, Hangzhou, China;; c The Key Laboratory of Conservation Biology for Endangered Wildlife of the Ministry of Education, and Laboratory of Systematic and Evolutionary Botany and Biodiversity, College of Life Sciences, Zhejiang University, Hangzhou, China

**Keywords:** Asparagaceae, *Barnardia*, chloroplast genome, phylogenomics

## Abstract

*Barnardia japonica* is an ornamental bulb with important medicinal usage. The complete chloroplast genome of *B. japonica* was newly sequenced in this study. The total chloroplast genome size of *B. japonica* was 156,129 bp. In total, 131 genes were identified, including 85 protein-coding genes, 8 rRNA genes, and 38 tRNA genes. Eighteen genes are containing introns (*clpP* and *ycf3* contained two introns) and 18 genes had two copies. The overall GC content of this genome was 37.7%. A further phylogenomic analysis of Asparagales, including 36 taxa, was conducted for the placement of genus *Barnardia*. The complete plastome of *B. japonica* will provide a valuable resource for further genetic conservation, phylogenomic, and evolution studies in the genus and family.

Asparagaceae, according to the APG III system (APG III 2009), in the order of Asparagales, is a family of monocotyledonous flowering plants, including 114 genera with a total of ∼2900 known species. *Barnardia* Lindley, is a small genus of bulbous flowering plants in the family Asparagaceae, subfamily Scilloidea. Only two species were included in this genus, *Barnardia japonica* and *Barnardia numidica*, which are mainly distributed in East Asia and Northwest Africa, Sounthwest Europe (Ali et al. 2012). *B. japonica* is heavily cultivated as an ornamental bulb showing attractive and sophisticated flowers. It also has been used for a wide range of medicinal applications to treat the rheumatism, cardiac, urinary infection, dermatological, and hemorrhoid disease (Chang 2017; Kayıran and Özkan 2017). Here, we assembled and characterized the first complete plastome of genus *Barnardia.* It will provide potential genetic resources for further evolutionary studies of the genus and other relatives in Asparaaceae.

Total DNA was extracted from fresh leaves of *Barnardia japonica* individual using DNA Plantzol Reagent (Invitrogen, Carlsbad). It is collected from Mingang, Wenzhou, Zhejiang, China (27°56'32.41"N, 120°31'34.06"E, Voucher No. WH1708010624, deposited at Zhejiang University). The plastome sequences were generated using Illumina HiSeq 2500 platform (Illumina Inc., San Diego, CA). In total, about 14.5 million high-quality clean reads (150 bp PE read length) were generated with adaptors trimmed. The CLC de novo assembler (CLC Bio, Aarhus, Denmark), BLAST, GeSeq (Tillich et al. 2017), and tRNAscan-SE v1.3.1 (Schattner et al. 2005) were used to align, assemble, and annotate the plastome.

The full length of *Barnardia japonica* chloroplast genome (GenBank Accession No. MH287351) was 156,129 bp and comprised of a large single copy region (LSC with 85,284 bp), a small single copy region (SSC with 18,429 bp), and two inverted repeat regions (IR with 26,208 bp). The overall GC content of the *B. japonica* cp genome was 37.7% and the GC content in the LSC, SSC, and IR regions are 35.8, 31.3, and 42.9%, respectively. A total of 131 genes were contained in the cp genome (85 protein-coding genes, 8 rRNA genes, and 38 tRNA genes. Eighteen genes had two copies, which included 6 PCG genes (*ndhB*, *rpl2*, *rpl23*, *rps12*, *rps7*, and *ycf2*), 8 tRNA genes (*trnA-UGC*, *trnH-GUG*, *trnI-CAU*, *trnI-GAU*, *trnL-CAA*, *trnN-GUU*, *trnR-ACG*, and *trnV-GAC*), and all 4 rRNA species (*rrn4.5*, *rrn5*, *rrn16*, and *rrn23*). Among the protein-coding genes, two genes (*clpP* and *ycf3*) contained two introns, and other ten genes (*atpF*, *ndhA*, *ndhB*, *petB*, *petD*, *rpl16*, *rpl2*, *rpoC1*, *rps12*, *rps16*) had one intron each.

Thirty-six chloroplast genome of Asparagales were fully aligned with MAFFT v7.3 (Katoh and Standley 2013), and the maximum likelihood (ML) inference was performed using GTR + I + Γ model with 1000 bootstrap replicates with RAxMLv.8.2.1 (Stamatakis 2014) on the CIPRES cluster service (Miller et al. 2010). The result revealed that *B. japonica* was most closely related to members of genus *Milla* with the current sampling extent (Figure 1). The newly characterized *B. japonica* complete chloroplast genome will provide essential data for further study on the phylogeny and evolution of the genus *Barnardia* and the family Asparagaceae.

**Figure 1.**
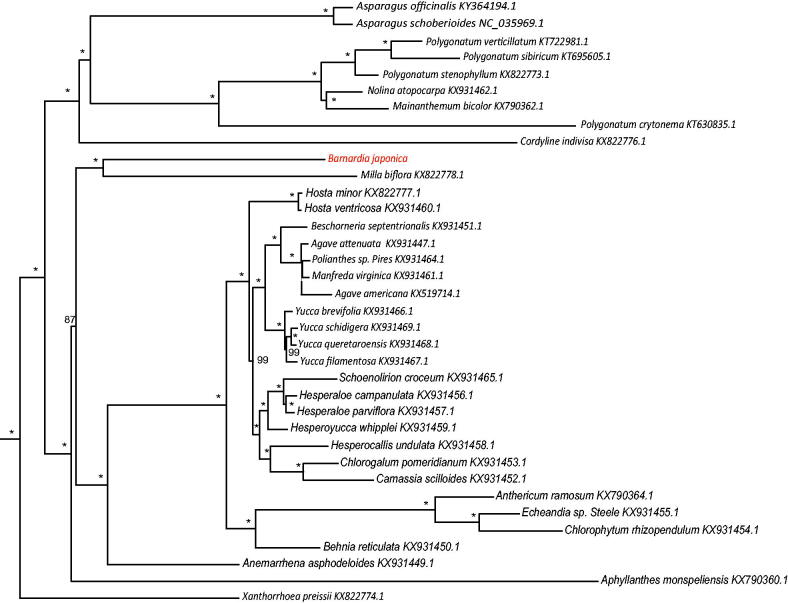
The best Maximum likelihood (ML) phylogram inferred from 36 chloroplast genomes in Asparagaceae (bootstrap value are indicated on the branches, ‘*’ denotes a fully supported node).
